# Development of a Sampling and Storage Protocol of Extracellular Vesicles (EVs)—Establishment of the First EV Biobank for Polytraumatized Patients

**DOI:** 10.3390/ijms25115645

**Published:** 2024-05-22

**Authors:** Birte Weber, Aileen Ritter, Jiaoyan Han, Inna Schaible, Ramona Sturm, Borna Relja, Markus Huber-Lang, Frank Hildebrand, Christiane Pallas, Marek Widera, Dirk Henrich, Ingo Marzi, Liudmila Leppik

**Affiliations:** 1 Department of Trauma-, Hand- and Reconstructive Surgery, University Hospital Frankfurt, Goethe University, 60486 Frankfurt am Main, Germanylleppik@yahoo.com (L.L.); 2 Translational and Experimental Trauma Research, Department of Trauma, Hand, Plastic and Reconstructive Surgery, University Hospital Ulm, 89081 Ulm, Germany; 3 Institute of Clinical and Experimental Trauma-Immunology, University Hospital Ulm, 89081 Ulm, Germany; 4 Department of Trauma and Reconstructive Surgery, University Hospital RWTH Aachen, 52074 Aachen, Germany; 5 Institute for Medical Virology, University Hospital Frankfurt, Goethe University, 60596 Frankfurt am Main, Germany

**Keywords:** polytrauma, size exclusion chromatography, freezing–thawing cycles, nanoparticle tracking, miRNAs, methodology, technical note

## Abstract

In the last few years, several studies have emphasized the existence of injury-specific EV “barcodes” that could have significant importance for the precise diagnosis of different organ injuries in polytrauma patients. To expand the research potential of the NTF (network trauma research) biobank of polytraumatized patients, the NTF research group decided to further establish a biobank for EVs. However, until now, the protocols for the isolation, characterization, and storage of EVs for biobank purposes have not been conceptualized. Plasma and serum samples from healthy volunteers (n = 10) were used. Three EV isolation methods of high relevance for the work with patients’ samples (ultracentrifugation, size exclusion chromatography, and immune magnetic bead-based isolation) were compared. EVs were quantified using nanoparticle tracking analysis, EV proteins, and miRNAs. The effects of different isolation solutions; the long storage of samples (up to 3 years); and the sensibility of EVs to serial freezing–thawing cycles and different storage conditions (RT, 4/−20/−80 °C, dry ice) were evaluated. The SEC isolation method was considered the most suitable for EV biobanking. We did not find any difference in the quantity of EVs between serum and plasma-EVs. The importance of particle-free PBS as an isolation solution was confirmed. Plasma that has been frozen for a long time can also be used as a source of EVs. Serial freezing–thawing cycles were found to affect the mean size of EVs but not their amount. The storage of EV samples for 5 days on dry ice significantly reduced the EV protein concentration.

## 1. Introduction

In 2013, a nationwide biobank of serum and plasma samples from polytraumatized patients was initiated as an expansion to the TraumaRegister DGU^®^ of the German Trauma Society [1]. This project was introduced to systemically evaluate and monitor polytraumatized patients in terms of their (patho-) physiological conditions and clinical treatment strategies. The aim was to establish a multicenter biobank with samples from polytraumatized patients, which should help to decrypt the complexity of the post-traumatic immune and organ response, to elucidate the underlying pathophysiology, diagnosis, and potential treatment targets for polytrauma patients. Currently, the NTF biobank represents a collaborative operational data and sample platform for numerous research purposes that is continuously fed by samples and the corresponding clinical data sets of polytraumatized patients from seven major trauma centers in Germany. In order to expand the biobank’s research potential, the DFG research group (FOR 5417) formed by members of the NTF aimed to expand the NTF biobank with extracellular vesicle (EV) samples. This expansion of the original serum/plasma biobank is the rational response to the emerging importance of the diagnostic and therapeutic potential of EVs in general and also in the field of trauma. EVs are generally characterized as small particles that are discharged by cells, enclosed by a lipid bilayer, and incapable of self-replication. The classification of extracellular particles has undergone and continues to undergo constant changes. Thus, the classification into exosomes, microvesicles, and apoptotic bodies is more of a historical approach, and it is no longer suggested for authors to categorize EVs into “small” (<200 nm) and “large” categories, as they did in the last few years. EVs have the ability to alter the phenotypes and functions of other cells by reflecting the condition of their parent cell through their complicated cargo [2]. In recent years, the number of publications focused on the role of extracellular vesicles as mediators and biomarkers of traumatic and non-traumatic injuries has significantly increased. As recently reviewed [3], several studies emphasized the possible existence of injury-specific EV “barcodes” (miRNA cargo or EV surface protein barcodes), which could have significant importance for the precise diagnosis of different organ injuries in polytrauma patients. These pioneer studies highlight the importance of the identification and verification of such EV barcodes. To identify new EV barcodes and EV biomarkers and to address their physiological role in inflammation and regeneration, a large number of patients and samples are needed. Therefore, a multicenter approach is mandatory.

A key prerequisite for the establishment of an EV biobank is the development of efficient and standardized isolation and storage methods, allowing the isolation of pure and homogeneous EV preparations in sufficient quantities. Despite the high number of published protocols addressing EV isolation and storage, none of them were revised with the focus of EV biobanking to the best of our knowledge. Criteria such as EV quantity and quality; the possibility to isolate miRNAs; the possibility to run multiple samples in parallel; and running time and costs were identified as key points in the process of generating a protocol suitable for nationwide biobanking. Also, the different equipment in the laboratory at different trauma centers is predicted while conducting a study to establish an EV isolation protocol with a focus on the biobanking aspect. In 2013, the International Society for EVs (ISEV) recommended preserving EV samples at −80 °C, further specifying that EVs should be stored in PBS in siliconized vessels [4]. However, later, in the updated version of 2018, standard indications for biological samples or EV storage were not provided anymore [5]. These guidelines, recognizing the impact of preservation on EVs and their matrix, suggest clearly describing the precise storage strategies and promoting further investigations to better clarify the effect of storage and thawing processes [6]. Besides the storage protocols, the question of the preferred isolation method is also not fully answered in the present literature. Ultracentrifugation is widely applied as the standard method to isolate EVs [7], although a few more alternatives are available, which enable the isolation of pure, uncontaminated EVs, thereby still being affordable.

## 2. Results

The aim of the present study was to establish a robust and reproducible EV isolation and storage protocol that is suitable for running a nationwide biobank of EVs obtained from polytraumatized patients.

First, we compared the performance of three different EV isolation methods thought to be applicable for EV biobanking. Criteria such as EV quantity and quality; the possibility to isolate miRNAs; the possibility to run multiple samples in parallel; and running time and costs were evaluated. The results of the comparison are summarized in Table 1.

As deduced from the table, the need for specific equipment, long running time, high sample volume, and low scalability makes the ultracentrifugation method unsuitable for EV biobanking purposes. Among the two other methods under comparison, SEC is preferential, as it does not limit the downstream applications of EV isolates [8]. Therefore, the following experiments were performed with SEC-isolated EVs.

**Figure 1:**
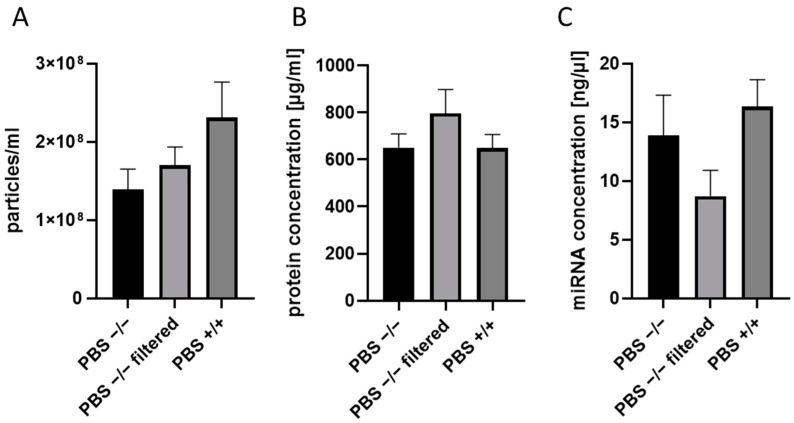
Effect of PBS solution content and purity on EV isolation with SEC. (**A**) Nanoparticle tracking analysis of EV isolates obtained with different PBS solutions (particles/mL). (**B**) Protein concentration of EVs isolates. (**C**) miRNAs concentration in EVs isolates. “PBS −/−” Dulbecco PBS without Ca/Mg; “PBS −/− filtered”—Dulbecco PBS without Ca/Mg filtered with 200 µL filter; “PBS +/+” Dulbecco PBS with Ca/Mg, not filtered. n = 5–10.

The SEC method requires the use of PBS solution (for column washing and EV elution), which, however, is not provided with the kit. One important step for the present analysis was the evaluation of which PBS solution should be used. We compared the performance of three different PBS solutions of the same manufacturer (Dulbeco, Sigma-Aldrich, Taufkirchen, Germany) and compared EV particle size distribution via NTA (Figure 1). No significant difference in the number of particles measured by NTA was observed; nevertheless, higher background signals were seen when unfiltered PBS solution or PBS (+/+) solutions were used (Figure 1A). The EV protein amount, as well as miRNA concentrations, were not influenced by the sort of PBS used (Figure 1B,C).

**Figure 2:**
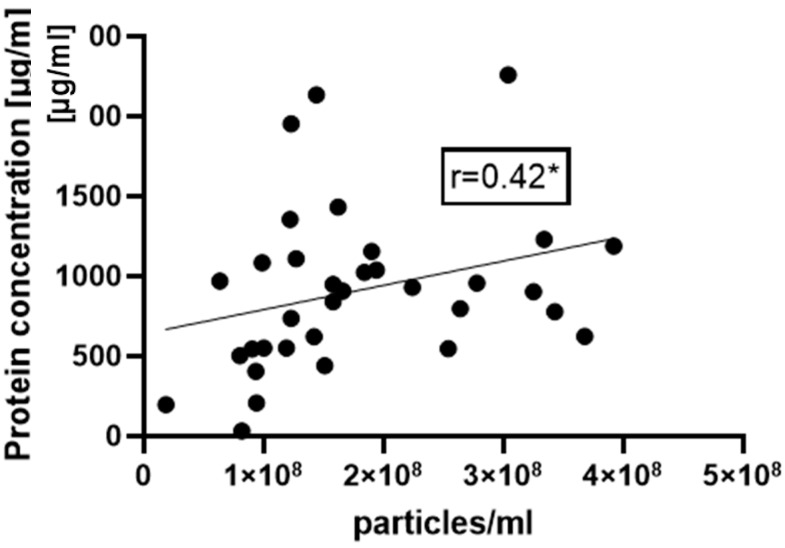
Number of particles measured in EV isolates with nanoparticle-tracking analysis moderately correlates with protein concentration of EV samples. r = 0.42, * *p* < 0.05, n = 35.

Although the use of the NTA method for the characterization of EV isolates sufficiently provides information about particle number and particle size distribution, it has the drawback of expensive equipment and time consumption. Therefore, it cannot be installed in each participating center. In contrast, EV quantification based on protein quantification (Bradford assay) belongs to the laboratory routine techniques and may be used as the initial method of EV isolate quantification. For verification, we quantified our EV isolates with both techniques and performed a correlation analysis of the results. A moderate correlation (r = 0.42, *p* < 0.05) was found between EV protein concentration and particle number measured by NTA (Figure 2).

**Figure 3:**
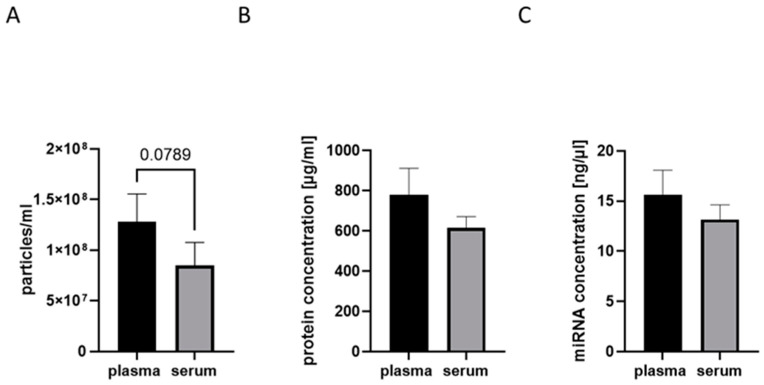
Comparison of plasma and serum as a source of EVs for EV biobank. (**A**) No significant differences in EV particle numbers were detected between serum and plasma samples of the same individuals, nanoparticle tracking analysis. (**B**) EV-protein and (**C**) EV–miRNA concentrations also do not significantly differ among plasma and serum EV isolates.

As the original NTF biobank incorporates both serum and plasma samples of polytrauma patients, it was important for us to analyze if both sources were suitable for EV biobanking (Figure 3). No significant differences, with regard to the number of particles (Figure 3A) and the EV protein (Figure 3B) and miRNA concentrations (Figure 3C) were detected between EV isolates from plasma and serum; however, a tendency towards lower values was frequently seen in serum samples.

**Figure 4:**
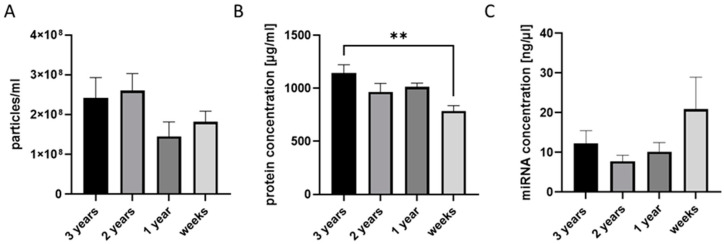
The storage of plasma for several years has no effect on the number and miRNA concentrations of isolated EVs. (**A**) The EV particle number measured by nanoparticle tracking (NTA). (**B**) EVs isolated from plasma stored for 3 years have higher protein concentrations than other EV isolates. (**C**) EV–miRNA concentrations do not differ among EV isolates. ** *p* < 0.01; n = 5.

As patient samples are always unique and limited in quantity, another important question for EV biobanking is the possibility of using samples that have been stored for a longer time period (several years) for EV isolation. Therefore, we compared the quality of EV isolates obtained from plasma samples stored for a few years (3 years) by means of EV particle numbers and EV protein and miRNA concentrations. We did not observe any significant difference in the number of EV particles measured by NTA (Figure 4A). However, the EV protein concentration was significantly higher in EVs isolated from corresponding plasma samples stored for 3 years (Figure 4B). No significant difference in EV–miRNA concentrations was found among the EV isolates (Figure 4C).

**Figure 5:**
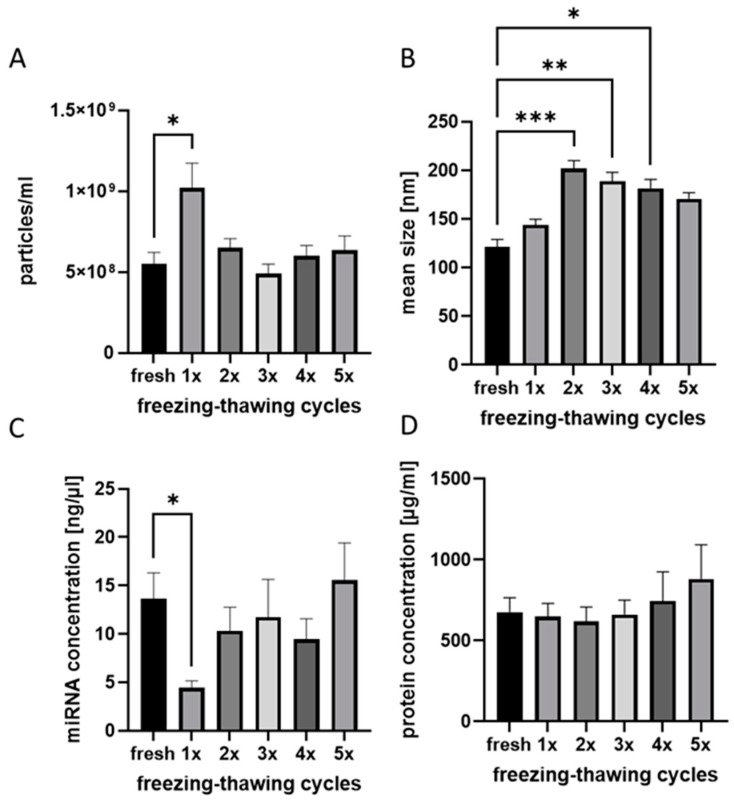
Effects of freezing–thawing cycles on plasma EVs isolates. (**A**) After one freezing–thawing cycle, EV isolates displayed higher EV particle numbers. (**B**) Freezing–thawing reveals an effect on EV size distribution. (**C**) miRNA concentration was significantly decreased in samples that were frozen once. (**D**) EV protein concentration was not affected by freezing and thawing. * *p* < 0.05; ** *p* < 0.01; *** *p* < 0.001, n = 10.

As the EV samples collected and stored in the biobank are expected to be used by multiple researchers and for different research purposes, it was important to characterize the optimal handling of EV probes in the biobank. We evaluated the effects of multiple freezing–thawing cycles on the EV samples’ quality and quantity (Figure 5).

No differences among EV particle numbers, measured in EV isolates that were freshly prepared or run through several freezing–thawing cycles, were found. The only exception was EV isolates, which were frozen and thawed once. These EV isolates were measured with significantly higher particle numbers compared to all other isolates (Figure 5A). At the same time, the freezing–thawing procedure was found to have an effect on the EV size distribution (Figure 5B). The size of EV particles increased significantly after the second freezing–thawing cycle as compared to non-treated (fresh) samples (Figure 5B). Similar to EV particle numbers, the EV’s miRNA concentrations in different EV isolates did not differ—with the exception of EV isolates after one freezing–thawing cycle (Figure 5C). In these samples, miRNA was significantly reduced. The EV protein concentration was not affected by freezing–thawing cycles (Figure 5D).

For the biobank, the exchange of samples between participating centers is an important part of collaborative efforts; therefore, proper sample-transportation conditions are highly relevant. Therefore, we evaluated the effects of the storage of EV isolates for 1 to up to 5 days at room temperature, +4 °C, −20 °C, −80 °C, and dry ice in a daily manner.

**Figure 6:**
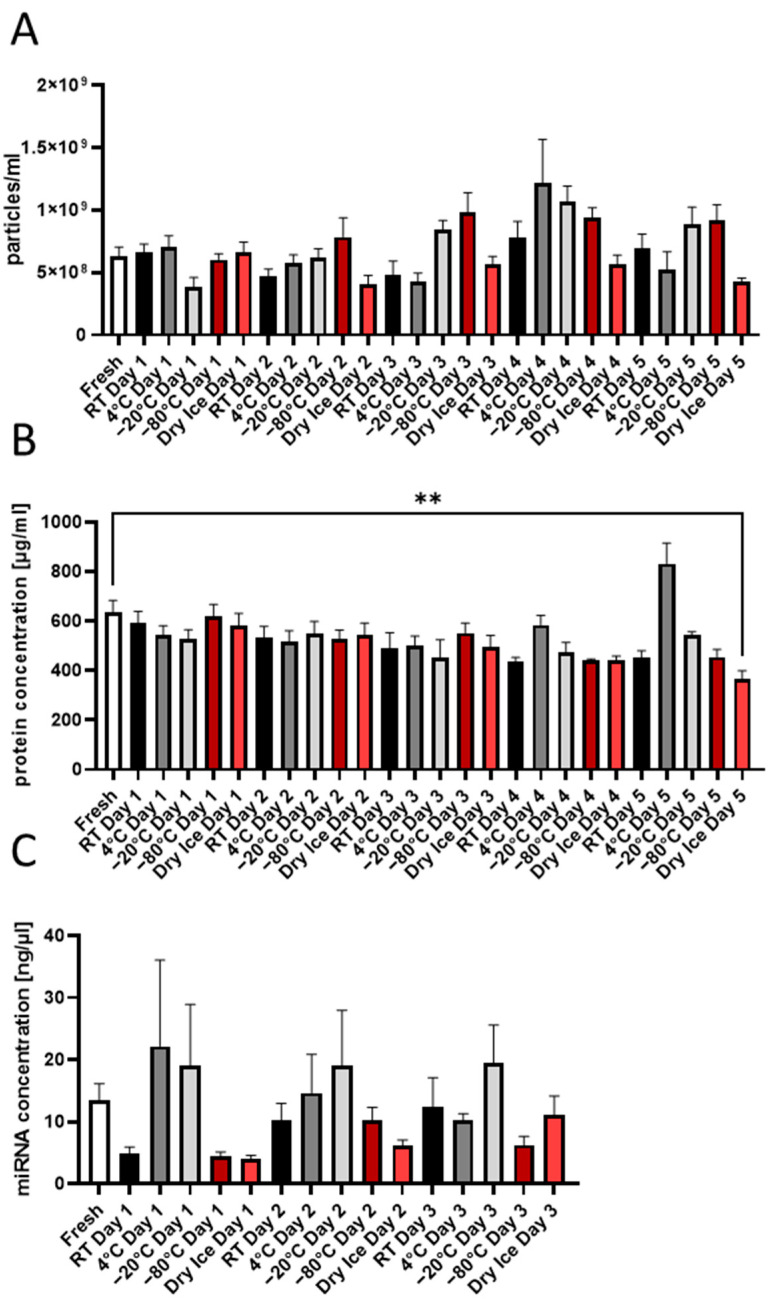
Effect of the storage temperature on EV particle number and Protein and miRNA concentrations. (**A**) Number of EV particles measured at days 0–5 of the storage at room temperature (RT), 4 °C, −20 °C, −80 °C, and dry ice. (**B**) The influence of the storage temperature on the EV protein concentration over the first 5 days. (**C**) The EV miRNA concentration over the storage time at room temperature (RT), 4 °C, −20 °C, −80 °C, and dry ice. ** *p* < 0.01; n = 5.

As presented in Figure 6, the storage of EV isolates up to the fifth day at different temperatures exhibited no effect on EV particle amount (Figure 6A), as well as on EV protein and RNA concentrations (Figure 6C). The only effect was observed in samples stored for 5 days on dry ice—these EVs isolates were characterized by a significantly lower protein concentration (Figure 6B).

## 3. Discussion

The processing and storage of biospecimens are crucial steps in any biobank development. Even though there are numerous studies evaluating various EV isolation techniques, none of them has specifically examined the protocol for EV isolation from patient blood material for biobanking purposes. Such a protocol, optimized to work with small quantities of starting material (e.g., patient’s plasma/serum) and large numbers of samples, should be simple enough to be easily integrated and transferred between research centers. Therefore, the present study focused on the development of an EV isolation and storage protocol serving as the base of the new NTF EV biobank. Various parameters, including an optimal EV isolation method, starting material (plasma, serum, long-term stored plasma), and optimal transportation/storage conditions were evaluated.

### 3.1. EV Isolation

Out of multiple EV isolation methods, we compared the use of ultracentrifugation, size-exclusion liquid chromatography (SEC), and an immuno-magnetic bead-based method to isolate EVs. Ultracentrifugation was chosen as a historical gold standard method for EV isolation, which hitherto is still the most commonly used technique [9]. Two other methods were additionally chosen as relatively fast assays using a minimal amount of body fluids [10]. Our results (summarized in Table 1) revealed that ultracentrifugation as a method has some significant limitations when used in EV biobanking due to its time-consuming nature and requirement for special and expensive equipment. The employment of immuno-magnetic bead-based approach in EV biobanking will strictly limit the number of downstream EV applications due to the presence of the magnetic beads in EV isolates. At the same time, SEC exhibited the best performance for our biobanking purposes. This user-friendly method allows us to work with a large number of small samples and is efficient (i.e., inexpensive), easily conductible without the need for special equipment, and easily adaptable to most laboratories. In the literature, SEC was described as a reproducible, scalable, and inexpensive EV isolation method that provides pure and functional EVs [11,12]. Importantly, it was demonstrated that SEC-isolated EVs retained their biological activity and integrity while being free of other soluble proteins such as lipoproteins (HDL) and plasma proteins [13,14,15]. This fact makes SEC-isolated EVs ideal samples for the search for new EV-associated biomarkers [16,17,18,19]. When developing such a protocol, a careful compromise between the purity of the final EV isolates and requirements like running concurrently in several clinical centers, processing many samples of low sizes, and yielding EV isolates with the widest possible range of potential downstream applications has to be found. This goal restricts the range of possible methods (particularly approaches based on the combination of methods) for EV isolation. It does not, however, limit the ability to further purify or alter the samples taken from the biobank in accordance with the requirements of any particular study.

In the present study, we propose the suitability of SEC-isolated EVs for polytrauma research and showed that such EVs could be used in a differential analysis of EV surface epitopes [20,21] and/or a cargo analysis of cytokines [22] and miRNAs [23]. It is known that isolation methods can influence the measured total protein content of EVs [24], as well as the EV-particle number [25]. According to the findings of our earlier study [4], which employed the same EV isolation protocol and comparable material (plasma from polytrauma patients and healthy volunteers), SEC-isolated EVs are CD9-, CD63-, and CD81-positive and can be quantified by EV protein concentration and NTA [20]. Interestingly, the NTA- and protein-quantification measurements were moderately correlated (as demonstrated in Figure 2). This might help to simplify the sample handling in the near future because protein concentration measurement belongs to the routine measurements in most laboratories, while NTA requires special equipment, is time-consuming, and is more complex to perform. Nevertheless, it is known that SEC leads to the co-isolation of albumin together with EVs, which might be one important limitation when working with albumin-rich plasma or serum samples [26].

In general, detecting and controlling the presence of possible co-isolated contaminants is of absolute importance for EV research and EV biobank. In regard to potential biomarkers, it is crucial that a certain biomarker or functional characteristic is connected to vesicles and not to co-isolated contaminants. There are several possibilities to address this issue; however, none of them seemed to be optimal for the EV biobank. Thus, electron microscopy (EM) analysis could detect large non-vesical aggregates, but this approach is inappropriate for regular use since not all trauma centers have easy access to EM. The detection of proteins that are not to be expected in EVs by means of Western blotting could be partially helpful, but the selection of such exclusion protein markers is challenging since we still do not have full knowledge of EV epitopes and cargo proteins, which may vary significantly between the patients’ groups. An interesting alternative could be the ratio of nano-vesicle counts to protein concentration, suggested by Webber et al. [27], as a “general, simple, and quantitative” parameter to estimate and compare EV sample purity that has been validated by authors with cell culture EVs. In future studies, this ratio parameter could be investigated in the plasma/serum EV samples obtained from a wide range of patients and validated with a broad range of potential contaminants. This might be a realistic standardization parameter of protein contaminations in EV biobanking.

In general, the area of EV research in modern science is highly broad, spanning from basic science to biomarker research and therapeutic applications. It is in permanent growth, progressing through the creation of new techniques and the acquisition of new knowledge. As in any other field of science, following the best practice and avoiding heterogeneity in nomenclature, characterization methods, and reporting procedures is of absolute importance for making further progress. Significant efforts were performed in order to provide “recommendations and guidance on EV-related studies”, summarized first in “Minimum Information for Studies of Extracellular Vesicles” (MISEV) [28] with later updates in MISEV2018 [5] and MISEV2023 [2]. The updated MISEV guidelines were implemented in the NTF EV biobank protocol regarding nomenclature, sample collection and pre-processing, SEC isolation, the characterization of EVs, and reporting (EV-TRACK assignment). We believe that the development of a nationwide EV biobank following MISEV guidance, together with future research, utilizing EV biobank samples, and adhering to the actual MISEV standards could significantly enhance EV research. All in all, future studies are needed to evaluate in detail the correlation of EV protein concentration and EV numbers, as well as the effects of co-isolation of albumin in patients’ samples.

As the next question, we addressed whether the use of plasma or serum for EV isolation should be preferred in the context of establishing an EV biobank. We did not find any significant difference in the EV numbers and EV protein and miRNA concentrations among EVs isolated from both sources. By trend, the serum isolates exhibited a reduced EV concentration, which should be confirmed in further studies. In the literature, it was reported that the overall miRNA content appears higher in plasma EVs compared to serum EVs. Interestingly, the total miRNA concentration was found to be higher in plasma EVs, while also more miRNAs from non-vesicle origins were detected in these samples. Therefore, the authors concluded that serum samples are more suitable for EV biomarker studies than plasma samples [29]. While dealing with the question of whether plasma or serum samples should be used, the effects of the anticoagulants used during plasma collection were also evaluated in several studies [30]. Thus, Karimi et al. (2022) found a higher number of CD9+ EVs present in EDTA plasma as compared to acid-citrate-dextrose plasma and serum. CD63+ EVs were enriched in serum, while CD81+ vesicles were the rarest subpopulation in both plasma and serum samples of healthy volunteers [31]. To increase the potential size and quality of future studies for our NTF EV biobank, we chose to gather EVs from serum as well as plasma. We selected clinically established EDTA as the anticoagulant for plasma collection because our NTF plasma biobank routinely uses EDTA as an anticoagulant for the systemic samples, which will allow the samples from both biobanks to be comparable.

We also evaluated if long-term (few years) storage at −80 °C yields negative effects on the quantity and quality of EV isolates. According to the present data, there is no difference in EV numbers or EV protein and miRNA concentrations among EV isolates gained from plasma samples that are freshly prepared or stored for up to 3 years. One limitation of these results is the fact that we did not use exactly the same healthy individuals over the years. This might influence the results. All in all, this important observation allows us to conduct retrospective studies and expand the number of EV samples for the biobank by using older plasma samples as a source. Since non-EV lipid particles including chylomicrons and lipoprotein particles are known to be abundant in human serum, they typically appear as contaminants in blood EV isolates. The clearing of these particles was not included in the developed protocol due to technical and methodological complexity; however, it is important to be aware of these contaminants when biobank EV isolates are planned to be used in biomarker development studies. Additional clearing steps [32] could be added, when relevant, in each individual study.

#### EV Storage and Transportation

Storage stability is a critical issue that needs to be resolved to enable the stability of biobanked clinical samples and the production of reliable EV isolates for research. Studies that analyzed how storage conditions affect EVs have shown that a variety of EV characteristics, such as biophysical stability, particle number and size, and function of EVs, may be impacted by storage-related factors [33,34,35,36,37,38,39].

Our own data and data from others demonstrated the importance of using particle-free media for isolation and dilution (e.g., 0.22 µm-filtered PBS) in order to avoid high background noises by NTA [40,41]. Concerning the EV storage media, temperature, and duration, there is a wide discrepancy in the literature that might be explained by differences in EV sources and isolation techniques among the studies. For example, Görgens et al. described that storing EVs in PBS at any temperature led to a drastic reduction of EVs over time and suggested the use of PBS supplemented with human albumin and trehalose for storage purposes of EVs [42]. Unfortunately, this could affect the downstream analysis of EV isolates with regard to protein analysis (e.g., Western blotting) and biomarker search. At the same time, Wu et al. described that for functional analysis, EVs could be stored at +4 °C or −20 °C for short-term preservation, and long-term preservation should be conducted at −80 °C [43]. Furthermore, the use of liquid nitrogen was found to be damaging for the EVs [43]. We analyzed the effect of short-term storage (5 days) of EV samples at different temperatures and conditions, as it is important for the logistics and transportation of the NTF EV biobank among participating research centers. We found the only negative effect in the case of storage in dry ice for 5 days. Gelibter et al. described that −80 °C storage reduced EV concentration and sample purity in a time-dependent manner while comparing freshly prepared samples vs. samples stored for 4 weeks vs. samples stored for 6 months. Furthermore, they found an increase in particle size after storage at −80 °C. Freezing–thawing cycles (max. three cycles, 1 week in between) led to a reduction in EV numbers after the first cycle and a cycle-dependent increase in particle size due to fusion phenomena during storage. Therefore, these authors recommended processing EVs from fresh, non-archival isolates [6]. We also analyzed the effects of freezing–thawing cycles on EVs and observed an increase in EV size already after the second cycle, which could be explained by EV fusion phenomena. Based on our results and literature analysis, for our exemplary NTF EV biobank, we decided to store EV samples in particle-free PBS at −80 °C in serial aliquots in order to minimize freezing–thawing cycles.

## 4. Materials and Methods

All analyses were performed with ethical approval given by the Local Ethics Committee of the University of Frankfurt (approval ID 89/19). Blood samples from healthy controls (n = 5–10) were taken and immediately kept on ice, and EDTA plasma or serum was gained by centrifugation at 3500 rpm and 4 °C for 15 min.

### 4.1. Isolation of EVs by Ultracentrifugation

Plasma/serum samples (500 µL) were cleared first by 10 min centrifugation at 2000× *g* and 4 °C and then by 30 min centrifugation at 10,000× *g* and 4 °C (Centrifuge 5427R, Eppendorf, Hamburg, Germany). The volume of cleared samples was increased to 40 mL (due to the working volume of the ultracentrifuge) in open-top polypropylene tubes (Beckman Coulter, Krefeld, Germany), and EVs were pelleted by 2 h centrifugation at 100,000× *g*, 4 °C (SW 32 Ti Rotor, Optima XPN-80 Centrifuge, Beckman Coulter, Krefeld, Germany). Pellets were washed with 1 mL 0.22 µM filtered Dulbecco’s PBS (−/−) (1× DPBS, Gibco^TM^, Sigma-Aldrich, Taufkirchen, Germany), precipitated by 1 h centrifugation at 100,000× *g*, 4 °C, and resuspended in 500 µL 0.22 µM filtered Dulbecco’s PBS (−/−).

### 4.2. Isolation of EVs by Size Exclusion Chromatography

EVs were isolated from 100 µL plasma or serum from healthy controls by size exclusion chromatography (Exo-Spin^TM^, Exosome Size Exclusion Column, Cell Guidance Systems, Cambridge, UK) according to the manufacturer protocol. For all experiments (except the analysis of isolation mediums), all washing and elution steps were performed with 0.22 µM filtered Dulbecco’s PBS (−/−). In case of the analysis of isolation media, EVs were isolated from plasma of five healthy controls, either by using filtered Dulbecco’s PBS (−/−), non-filtered Dulbecco’s PBS (−/−), or non-filtered Dulbecco’s PBS with Ca^2+^ and Mg^2+^ (PBS (+/+), 1× DPBS, Calcium, Magnesium; Gibco^TM^, Sigma-Aldrich, Taufkirchen, Germany). A short version of the NTF EV biobank isolation and storage protocol based on this method is included as Appendix A in this manuscript. We have submitted all relevant data of our experiments to the EV-TRACK knowledgebase (EV-TRACK ID: EV240047) [44].

### 4.3. Isolation of EVs by Immuno-Magnetic Bead-Based Method

EVs were isolated from 500 µL plasma/serum by using the Exosome Isolation Kit Pan (Miltenyi Biotec, Bergisch Gladbach, Germany) according to the manufacturer’s instruction.

### 4.4. Measurements of EV Protein Concentration

EV protein concentration was measured with Coomassie Plus (Bradford) Assay (Thermo Fisher Scientific, Rockford, IL, USA). Briefly, 10 µL of EV isolates were mixed with 300 µL Coomassie Plus reagent and incubated for 10 min, and absorbance was measured at 595 nm. Protein concentration was calculated with the help of calibration curve made with serial dilutions of protein standard.

### 4.5. EV–miRNA Isolation and Quantification

miRNAs were isolated from 150 µL of plasma EVs using the miRNeasy serum/plasma kit (Qiagen Inc., Hilden, Germany) according to the manufacturer protocol. For each sample, miRNA concentration was assessed spectrophotometrically (NanoVue Plus, GE Healthcare, Frankfurt am Main, Germany) in triplicate, and mean values were calculated.

### 4.6. EV-Nanoparticle Tracking Analysis (NTA) Assay

EV particles’ number and size distribution were determined by NTA (Nanosight NS500, Malvern Panalytical, Kassel, Germany). A total of 10 µL of isolated plasma EVs was diluted 1:100 in 0.22 µM filtered Dulbecco’s PBS (−/−). Capturing options were set to 60 s, and six replicates were performed. Particles were tracked under constant flow, and the mean value of the six replicates was used for the statistical analysis. For background measurements, 0.22 µM filtered Dulbecco’s PBS (−/−), Dulbecco’s PBS (−/−), and Dulbecco’s PBS (+/+) were measured using the same conditions (Appendix B and Supplementary Figures S1 and S2).

### 4.7. Effect of Long Plasma Storage on EVs

EVs were isolated via SEC from plasma samples of healthy volunteers (not the same individuals over time), which were collected, frozen, and stored at −80 °C for 1, 2, and 3 years or samples taken weeks ago (for each year n = 5). The plasma samples did not run through freezing–thawing cycles prior to the experiment. The number of EVs was quantified via NTA, and the EV proteins and miRNAs were quantified as described before and compared among the groups.

### 4.8. Effect of Freezing–Thawing Cycles on EVs

EVs were isolated via SEC from the plasma of 10 healthy volunteers, and then the EV particle number, proteins, and miRNAs were quantified directly after isolation. Isolates of EVs were run through serial cycles (1–5) of freezing–thawing as follows: EVs isolates were frozen at −80 °C for a minimum of 24 h and then fully thawed at room temperature. The EV particle number and miRNA and protein concentrations were assessed after each cycle.

### 4.9. Effect of Different Storage Conditions on EVs

EVs were isolated from plasma of healthy volunteers (n = 5–10) via SEC and isolates were stored for up to 5 days at either room temperature, 4 °C, −20 °C, −80 °C, or on dry ice. The EV particle numbers and EV protein and miRNA concentrations were measured daily for 4 consecutive days.

### 4.10. Statistical Analysis

GraphPad Prism 9 (Dotmatic, San Diego, CA, USA) was used for all statistical analysis. Data were analyzed by Kruskal–Wallis test followed by Dunn’s multiple comparison test. Correlation analysis was carried out with the Spearman rank correlation. Results with *p* ≤ 0.05 were considered significant. Data are presented as mean ± standard error of the mean (SEM).

## 5. Conclusions

We developed an effective and efficient isolation and storage protocol for the first biobank of EVs from polytraumatized patients. In the newly established NTF EV biobank, EVs should be isolated from patients’ serum and plasma via SEC with the use of particle-free PBS. The transport of samples between centers should be performed in dry ice but should not exceed 4 days. 

## Figures and Tables

**Table 1:** Size exclusion chromatography appears as the preferred isolation method for the establishment of an EV biobank of polytraumatized patients.

Isolation Method	Ultracentrifugation	Size Exclusion Chromatography	Immuno-Magnetic Beads
Specific equipment	Ultracentrifuge Optima XPN-80; Rotor SW 32 Ti; (Beckmann Coulter, Krefeld, Germany)	-	Magnetic separator (µMACS^TM^, Miltenyi Biotec)
Specific reagent	-	Exosome purification Kit for blood sera (EX03, Cell Guidance System, Cambridge, UK)	Exosome Isolation KIT Pan human (Miltenyi Biotec, Bergisch Gladbach, Germany)
EV quantity and quality			
EVs (Protein in µg/mL)	<0.5	<15	<1
miRNAs (for RT-qPCR)	+	+	+
Detection of CD9/CD63/CD81 by western blot	+	+	+
Handling/technical aspects			
Run time	Several hours	<2 h	<2 h
Sample number limitation	Limited by centrifuge (max. 6 samples)	High scalability (96 well format available)	Limited by magnetic separator
Approx. costs (pro 500 µL plasma/serum sample)	Minimal (consumables)	<10 €	<20 €
Disadvantages	Sample dilution/high sample volume needed, special and expensive equipment necessary, time-consuming	Re-use of columns might influence the samples	Magnetic beads are present in EV isolates and might influence downstream analysis [8]

**Figure A1:**
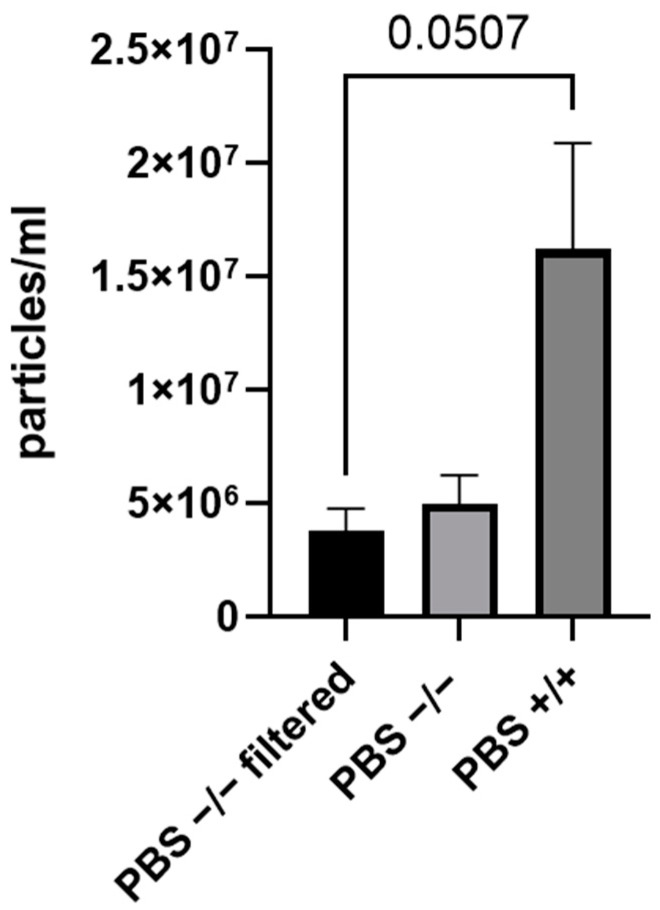
Comparison of Nanoparticle tracking analysis of filtered PBS, PBS −/− and PBS +/+ (Ca^2+^/Mg^2+^).

## Data Availability

The original contributions presented in the study are included in the article, further inquiries can be directed to the corresponding author/s.

## Supplementary Materials

The supporting information can be downloaded at: https://www.mdpi.com/article/10.3390/ijms25115645/s1.

## Appendix A

Short version of the NTF EV biobank isolation and storage protocol:

The protocol of the NTF-EV biobank is based on the Exo-Spin^TM^, Exosome Size Exclusion Column, (Cell guidance systems, Cambridge, UK). The first steps of the NTF EV biobank protocol are identical to the first steps in the protocol of the NTF Serum and Plasma Biobank the withdrawal of blood from polytraumatized patients (ISS > 25) in serum or EDTA tubes and centrifugation at 3500 rpm (2000× *g*) for 15 min and 4 °C to gain serum or plasma accordingly. Prior EV isolation another centrifugation step is necessary: 16,000× *g*, 30 min at 4 °C. For the following steps, the Exo-Spin^TM^ columns have to be prepared (according to the manufactures introduction) first by equilibration at room temperature for 15 min. The outlet plugs have to be removed before the Exo-Spin columns are inserted in a waste collection tube. Afterwards, the buffer in the columns has to be aspirated and discarded. During the whole process it is important to prevent the column from drying by immediate processing to the next steps. The columns should be washed twice with 250 µL of filtered PBS (0.22 µM filter). Afterwards, 100 µL of the previously prepared serum/plasma samples should be loaded onto the column. EVs are eluted from columns with 180 µL of filtered PBS (0.22 µM filter) into a fresh collection tube. The collected EVs should be briefly centrifuged at 100× *g*, aliquoted and frozen at −80 °C. The columns can be re-used up to five times, after four times washing with 250 µL of filtered PBS. Nevertheless, the re-use of the columns should be documented on the columns/in the Material and Method section and is not intended for biobanking. For the handling of the NTF EV biobank samples freezing and thawing cycles should be avoided as good as possible. The transport of EV samples is possible on dry ice, but should not exceed 3 days.
