# Supplementary material for: Development of a Sampling and Storage Protocol of Extracellular Vesicles (EVs)—Establishment of the First EV Biobank for Polytraumatized Patients
Source: Int J Mol Sci. 2024 May 22;25(11):5645. doi: 10.3390/ijms25115645 (PMC11172154; doi:10.3390/ijms25115645)
Supplement: Supplementary file 1 [file ijms-25-05645-s001.zip › ijms-2951781-supplementary.pdf]

Development of a Sampling and Storage Protocol of Extracellular Vesicles (EVs) - Establishment of the First EV-Biobank for Polytraumatized Patients

Birte Weber [1*], Aileen Ritter [1], Jiaoyan Han [1], Inna Schaible [1], Ramona Sturm [1], Borna Relja [2], Markus Huber-Lang [3], Frank Hildebrand [4], Christiane Pallas[5], Marek Widera[5], Dirk Henrich [1], Ingo Marzi [1] and Liudmila Leppik [1]

Supplementary data.

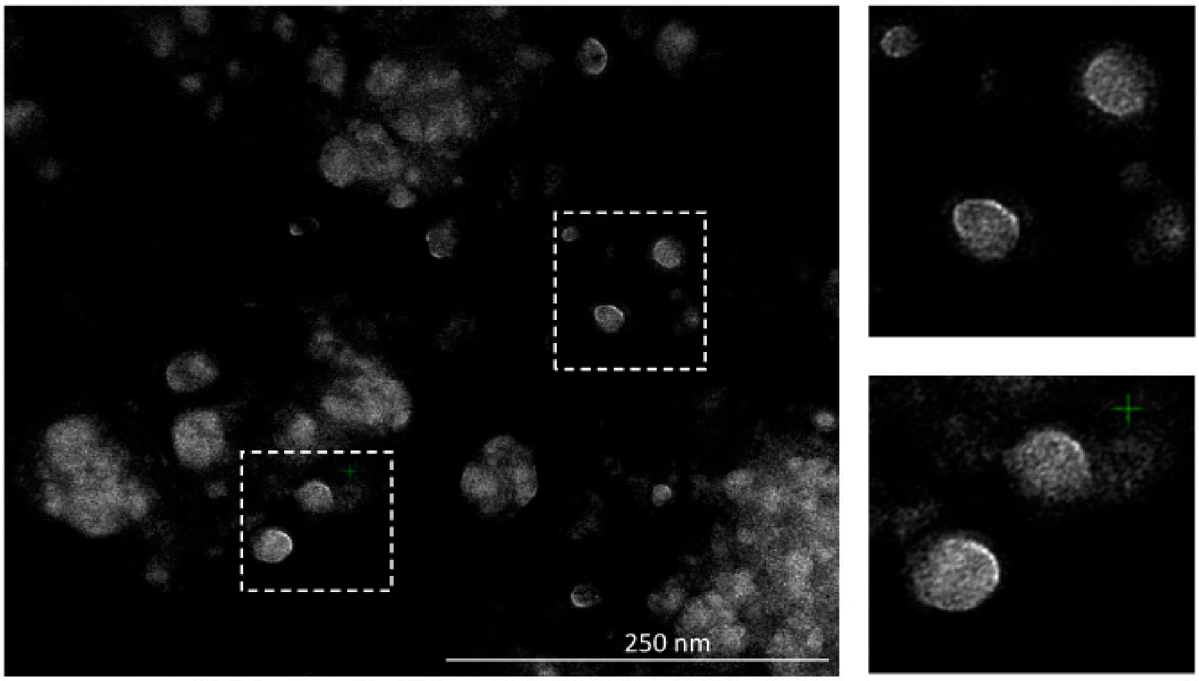

Figure S1: Transmission electron microscopy images of representative EV sample. Transmission electron microscopy (TEM) staining was performed according to standard diagnostic procedures. Briefly, grids were prepared by immersion in 1% Alcian blue solution followed by multiple rinses in distilled water. EV samples were then transferred to the prepared grids and incubated at room temperature for 10 min, followed by multiple washes with distilled water. Staining was performed with 3% phosphotungstic acid and 2% uranyl acetate solution. The grids were gently placed on filter paper to remove excess solution, then dried and subjected to TEM analysis using a Zeiss EM900 TEM instrument. Scale bar=250 nm.

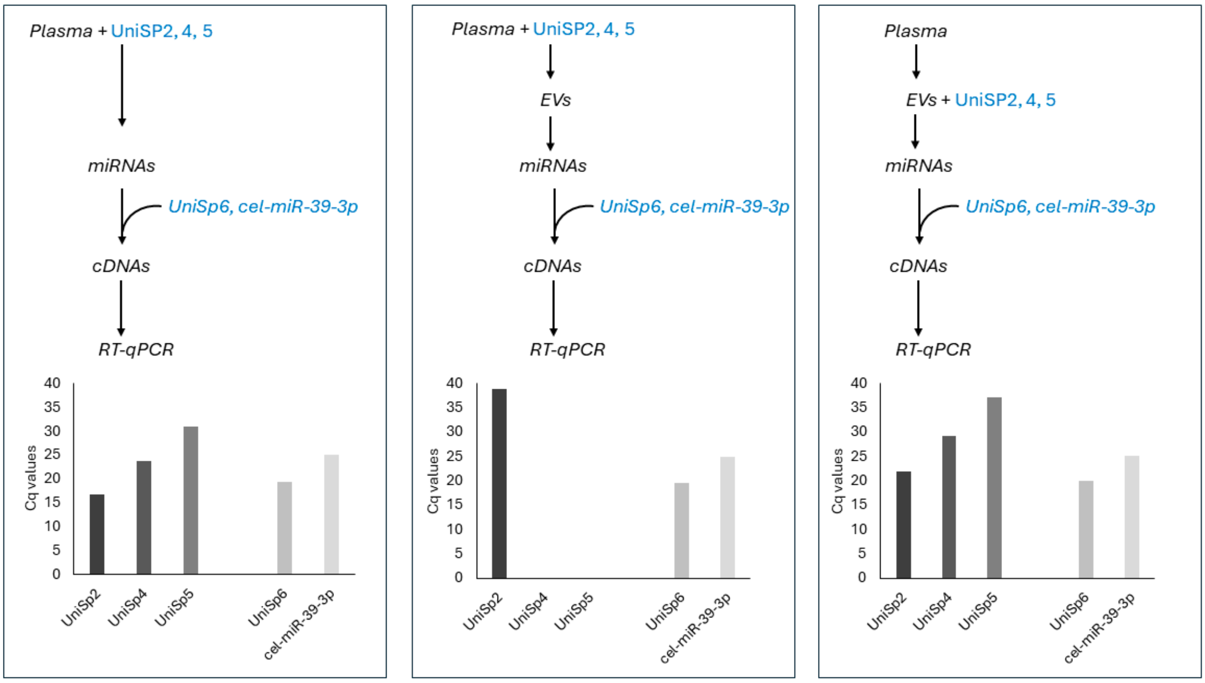

Figure S2: EV isolates have negligible plasma -miRNAs contamination.

Two plasma samples (each 200 µl) were mixed with three spike-In miRNAs (concentrations: UniSp2=160 fmol, UniSp4=1.6 fmol, UniSp5=0.016 fmol, Qiagen). These plasma samples were either directly (left panel) used for miRNAs isolation with miRNeasy Serum/Plasma advanced Kit (Qiagen), or first EVs were isolated (Exo-spin™, Cell Guidance) and then miRNAs were isolated (middle panel). As a control (right panel), 200 µl plasma sample was first proceeded for EVs isolation, then spike-Ins were added to EV isolates and miRNAs were isolated. All obtained miRNAs were proceeded for cDNA synthesis with UniSp6 and cel-miR-39 spike-Ins with miRCURY LNA RT Kit (Qiagen). All spike-Ins were detected by mean of RT-qPCR and results are shown as Cq values. Only UniSp2 miRNAs still could be detected in EV isolates (middle panel), however 22 cycles later as in plasma sample (left panel).
